# Cultural differences in on-line sensitivity to emotional voices: comparing East and West

**DOI:** 10.3389/fnhum.2015.00311

**Published:** 2015-05-29

**Authors:** Pan Liu, Simon Rigoulot, Marc D. Pell

**Affiliations:** ^1^School of Communication Sciences and Disorders, Centre for Research on Brain, Language, and Music (CRBLM), McGill UniversityMontréal, QC, Canada; ^2^International Laboratory for Brain, Music and Sound Research (BRAMS), Centre for Research on Brain, Language, and Music (CRBLM), McGill UniversityMontréal, QC, Canada

**Keywords:** cross-cultural, EEG/ERPs, vMMN, facial expression, emotional prosody

## Abstract

Evidence that culture modulates on-line neural responses to the emotional meanings encoded by vocal and facial expressions was demonstrated recently in a study comparing English North Americans and Chinese (Liu et al., 2015). Here, we compared how individuals from these two cultures passively respond to emotional cues from faces and voices using an Oddball task. Participants viewed in-group emotional faces, with or without simultaneous vocal expressions, while performing a face-irrelevant visual task as the EEG was recorded. A significantly larger visual Mismatch Negativity (vMMN) was observed for Chinese vs. English participants when faces were accompanied by voices, suggesting that Chinese were influenced to a larger extent by task-irrelevant vocal cues. These data highlight further differences in how adults from East Asian vs. Western cultures process socio-emotional cues, arguing that distinct cultural practices in communication (e.g., display rules) shape neurocognitive activity associated with the early perception and integration of multi-sensory emotional cues.

## Introduction

Communicating our feelings with one another is an integral part of human life, one that commonly involves two nonverbal information channels: facial expression and vocal expression (Grandjean et al., 2006; Paulmann and Pell, 2011). With increasing globalization, the context for inter-personal communication frequently involves people from different cultural backgrounds, which can sometimes lead to misunderstandings and conflict. Therefore, achieving a better understanding of cultural differences in communication is a laudable goal for scientific research that could benefit inter-cultural relations in the real world.

In day-to-day interactions, emotion processing occurs in various social environments and under different levels of awareness; in some instances, people attentively focus on another’s emotional expressions to actively discern their meaning. In others, emotional cues are detected when people are not purposely attending to them (Schirmer et al., 2005; Paulmann et al., 2012), but nonetheless used to construct a representation of how a social partner feels (Pell et al., 2011). A small literature now demonstrates that cultural background has a significant impact on how participants use facial and vocal cues to *consciously* evaluate the emotional meanings of these expressions from multi-sensory stimuli (Tanaka et al., 2010; Liu et al., 2015). Using an emotional Stroop-like task, Liu et al. (2015) compared behavioral responses and event-related brain potentials (ERPs) for two cultural groups, English-speaking North Americans and Chinese. Each group performed separate conditions where they judged the emotional meaning of a static face (sad or fearful) while ignoring a concurrent voice, or* vice versa*. The emotions of the face and voice were either congruent or incongruent, presented simultaneously in each trial for 800 milliseconds (ms); only culturally-appropriate (“in-group”) stimuli were judged by each group. Results indicated that both groups were sensitive to the congruence of the emotion communicated in the to-be-ignored channel (with lower accuracy and larger N400 amplitude for incongruent vs. congruent face-voice pairs). More critically, marked group differences were observed in how emotional congruence affected both accuracy and N400 responses; when attending to the voice, English participants showed much greater influence from to-be-ignored faces than Chinese participants. These results suggest that in comparison to Chinese, North Americans (or more broadly, individuals from Western cultures) are more sensitive to facial expressions than to vocal cues during emotion processing. Moreover, they underscore for the first time that cultural differences manifest not only in behavior but in the on-line neural semantic processing of emotional cues, as indexed by N400 (Liu et al., 2015). This claim complements and extends behavioral work by Tanaka et al. (2010) showing that Dutch participants are more attentive to facial expressions when perceiving multi-channel emotional information, whereas Japanese participants—or perhaps individuals from East Asian cultures more generally when coupled with Liu et al. (2015) data—are more sensitive to vocal cues conveying emotion.

The observed cultural differences have been interpreted within the context of *display rules*, i.e., culture-specific social norms that regulate how emotions are communicated in socially appropriate ways (Ishii et al., 2003; Park and Huang, 2010; Engelmann and Pogosyan, 2013). In contrast to Western individualist cultures, it is said that East Asian collectivist cultures value harmonious social relations above all (Hall and Hall, 1990; Scollon and Scollon, 1995), thus adopting certain display rules to maintain harmony and prevent conflicts. These conventions include (Gudykunst et al., 1996; Matsumoto et al., 1998) restraining facial expressions (Ekman, 1971; Markus and Kitayama, 1991; Matsumoto et al., 1998, 2005, 2008), avoiding eye contact (Hawrysh and Zaichkowsky, 1991; McCarthy et al., 2006, 2008), and using more indirect expressions in speech, e.g., unfinished sentences or vague meanings (Bilbow, 1997). A possible impact of East Asian display rules is that facial and verbal-semantic cues tend to be less salient and/or available during inter-personal communication; rather, people learn to rely more on vocal cues to communicate their feelings (Liu et al., 2015). In contrast, individuals from Western cultures, which encourage facial expressivity, consider eye contact as polite and sincere; they tend to employ more direct speech and are more attuned to facial and semantic information during their social interactions (Kitayama and Ishii, 2002; Ishii et al., 2003; Tanaka et al., 2010). These distinct communicative practices, reinforced by years of culture-specific learning, may well contribute to differences in how East Asian and Western cultures attend to and assign meaning to socio-emotional cues encountered in different communication channels, with enduring effects on how the neurocognitive system operates at particular processing stages (Liu et al., 2015).

Although cross-cultural differences have been detected in tasks when participants explicitly attend to emotional meanings of the stimuli, many emotional signals are encountered when people are not paying attention to the stimuli. For example, in the course of giving a speech, the speaker may inadvertently perceive certain changes in the audience, such as a disapproving face or vocalization, which rapidly captures their attention and leads to a more in-depth social evaluation of these cues (Schirmer and Kotz, 2006). To understand whether culture plays a role in how emotion is processed from faces and voices outside of attentional control, an Oddball-like paradigm could prove highly instructive. Previous studies in which participants passively view a series of facial expressions, while performing a face-irrelevant task, show that infrequent changes of the facial emotion (deviant trials) elicit a more negative-going ERP component when compared to what is observed for frequent unchanged facial expressions (standard trials). The negative difference wave between deviant and standard trials is considered a visual Mismatch Negativity (vMMN), a neural index of the early passive detection of infrequent mismatching information in the visual modality (Susac et al., 2004; Zhao and Li, 2006; Astikainen and Hietanen, 2009).

In an important study that examined the MMN to investigate early integration of face and voice information about emotions in a single (Western) cultural group, de Gelder et al. (1999) conducted a face-voice Oddball task where participants always heard angry voices paired with angry or sad faces. Congruent pairs (*angry face-angry voice*) served as standard trials (85% of total trials) while incongruent pairs (*sad face-angry voice*) served as deviants (15%). Participants passively viewed the faces while ignoring the voices. The authors reported an auditory MMN (aMMN)-like component peaking at 178 ms for deviants relative to standards; as the auditory counterpart of visual MMN, aMMN represents a neural indication of the early passive detection of unattended rare changes in the auditory channel (Näätänen, 1992). Since auditory stimuli were identical across all trials (angry) in their study while facial expressions (angry or sad) were manipulated as deviants, differences in the aMMN were interpreted as referring to the facial channel that bore the deviant information (de Gelder et al., 1999). This suggests that initial, pre-attentive stages of cross-sensory emotion perception and integration take place prior to 200 ms post-stimulus onset (Pourtois et al., 2000; Jessen and Kotz, 2011). The possibility that cultural factors somehow modulate brain responses to emotional stimuli at this early processing stage has not been tested, although this could allow a finer look at whether Eastern and Western cultures fundamentally differ in the use of different sources of emotion cues in communication (Ishii et al., 2003; Tanaka et al., 2010; Liu et al., 2015).

Indeed, while it is unknown whether culture modulates the MMN in the context of emotion processing, there is affirmative evidence in the domain of color perception that this component is sensitive to *language* background of the participants. Using an Oddball-like task of blue-green color perception, Thierry et al. (2009) found that Greek participants, in whose language there are two distinct terms distinguishing *light blue* and *dark blue*, showed a larger visual MMN component than English participants in response to deviants of *dark blue* circles relative to standards of *light blue* circles (in English both colors are referred to as *blue* without distinction). These results argue that the vMMN in color perception was modulated by the cultural-language background of the Greek speakers, who exhibited an early sensitivity to the contrast between dark and light blue owing to specific characteristics of their language and their resulting effects on the neurocognitive system (Thierry et al., 2009). This small but growing literature provides a foundation for the current study of how culture shapes on-line neural responses to multi-sensory emotional stimuli as humans process these cues largely outside of conscious control, in an earlier time window than investigated previously (Liu et al., 2015), as indexed by the MMN.

Here, we adopted many of the methods described by Liu et al. (2015) in their cross-cultural study of emotional-meaning processing to test the hypothesis that culture affects even earlier stages of integrating face with voice information about emotions, using an Oddball-like paradigm similar to de Gelder et al. (1999). Two cultural groups, *English-speaking North Americans* and *Mandarin-speaking Chinese*, were compared using identical facial and vocal stimuli and the same participants who took part in our previous study. Based upon previous indications that: (1) East Asians are more attuned to vocal expressions than Westerners, whereas Westerners are more oriented towards facial cues than East Asians (Tanaka et al., 2010; Liu et al., 2015); (2) early emotion integration of face and voice cues modulates the MMN component (de Gelder et al., 1999); and (3) the vMMN is sensitive to linguistic variables in the domain of color perception (Thierry et al., 2009), we hypothesized that a vMMN component would be elicited by deviations in the facial expression for both groups (where the vocal expression remains constant across trials). However, we predicted that the Chinese group would be influenced to a larger extent than the English group by accompanying vocal cues; this enhanced vMMN component would exemplify the role of culture at an early stage of multi-sensory emotion integration. As related ERP data are largely lacking, these data would supply unique insights about the nature and temporal characteristics of cultural effects on the cortical response to multi-sensory emotional cues that are an integral part of human communication.

## Method

### Participants

The two groups of participants tested in our previous study (Liu et al., 2015) also completed the Oddball experiment. The first group consisted of 19 *English-speaking North American*s (10 female, 9 male; mean age = 25 ± 3.91 years; years of education = 14.18 ± 2.19 years). Each of these participants: (1) was born and raised in Canada or in the northeast U.S. and spoke English as their native language; (2) had at least one grandparent of British descent on both the maternal and paternal side, with all grandparents of Western European descent. The second group consisted of 20 *Mandarin-speaking Chinese* participants (10 female, 10 male; mean age = 24.55 ± 2.75 years; years of education = 16.45 ± 2.68 years), who were all born and raised in Mainland China as native Mandarin speakers and had lived out of China for *less than 1 year*. Mean years of age (*F*_(1,37)_ = 0.253, *p* = 0.618) and years of education (*F*_(1,37)_ = 1.039, *p* = 0.319) were matched between the two groups. No participant reported any hearing impairment, and all reported normal or corrected-to-normal vision. All participants gave informed written consent before participation, which was approved by the Institutional Review Board of the Faculty of Medicine at McGill University. All were financially compensated for their participation.

### Stimuli

**Figure 1:**
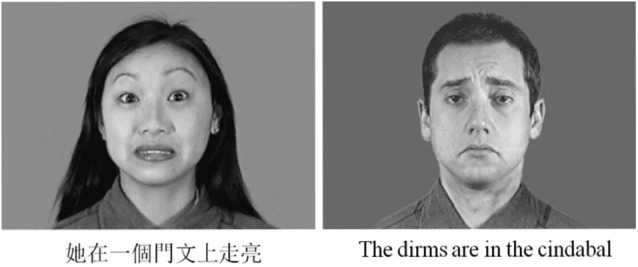
**Examples of facial and vocal stimuli**. Left, example of Chinese fearful face and Chinese pseudo-sentence. Right, example of Caucasian sad face and English pseudo-sentence.

This study employed the same facial and vocal stimuli as Liu et al. (2015). For the vocal stimuli, 8 pseudo-utterances (2 items × 4 speakers) spoken in sadness or fear were selected from validated recording inventories for Chinese (Liu and Pell, 2012) and English (Pell et al., 2009). Each recording was cut from the onset to a total duration of 800 ms to ensure consistent length across items. The peak amplitude of each recording in both groups has been normalized to 75 dB to mitigate gross differences in perceived loudness. Pseudo-utterances are composed of pseudo content words conjoined by real function words, rendering the utterances meaningless but possessing natural segmental/supra-segmental properties of the target language (Banse and Scherer, 1996; Pell et al., 2009; Rigoulot et al., 2013). In our studies, we chose to use emotional pseudo-utterances that resemble human speech, rather than non-linguistic vocalizations (e.g., crying), to better approximate what occurs in daily communication (see also de Gelder et al., 1999). Facial stimuli consisted of 6 black-white faces (1 item × 6 actors) expressing sadness or fear posed by Chinese or Caucasian actors (Beaupre and Hess, 2005). The two emotions, sadness and fear, were selected due to the strict selection criteria we adopted in our previous study to match the emotion recognition rates and emotional intensity of the stimuli between modalities (facial and vocal), emotions (sadness and fear), and groups (Chinese and English), to prevent stimulus-related confounds and permit valid cross-cultural comparisons (Table 1; for more details see Liu et al., 2015). Faces were synchronized with voices posed by the same cultural group to construct face-voice pairs of 800 ms, including both congruent (fearful face and fearful voice, sad face and sad voice) and incongruent (fearful face and sad voice, sad face and fearful voice) pairs. Only in-group face-voice pairs were presented to each group given our objective to understand emotion processing in the context of each group’s native culture and language (Figure 1).

**Table 1:** **Mean recognition rates (percent correct target identification) and emotional intensity ratings of the vocal and facial stimuli by emotion and cultural group (standard deviation in parentheses)**.

	Voices				Faces			
	English		Chinese		English		Chinese	
	Fear	Sadness	Fear	Sadness	Fear	Sadness	Fear	Sadness
*Recognition rates*	90.6 (1.8)	92.5 (2.7)	91.2 (5.2)	91.2 (5.2)	90.8 (8.6)	91.7 (9.3)	91.2 (7.6)	90.9 (10.0)
*Intensity ratings*	2.4 (0.3)	2.4 (0.4)	2.5 (0.3)	2.4 (0.3)	2.4 (0.3)	2.4 (0.4)	2.3 (0.4)	2.6 (0.5)

*For each stimulus, the participant made two consecutive judgments: they first identified the emotion being expressed by each item in a six forced-choice emotion recognition task (with happiness, sadness, anger, disgust, fear, neutrality as the 6 options); immediately after, they rated the intensity of the emotion they had selected in the previous recognition task on a 5-point rating scale, where 0 indicated “not intense at all” and 4 indicated “very intense”*.

In addition to the vocal and facial stimuli, two pure tone auditory stimuli lasting 800 ms were constructed to act as non-vocal auditory stimuli in one condition presented to each group (four pure tone stimuli total). The frequency of each pure tone stimulus was determined by calculating the mean fundamental frequency values of the fearful and sad vocal expressions produced by speakers of each language (Chinese fearful voices: 267 Hz, Chinese sad voices: 249 Hz; English fearful voices: 266 Hz, English sad voices: 187 Hz).

### Task and Design

An Oddball task composed of three experimental conditions was presented. In Condition 1, facial expressions were presented without any auditory stimuli to serve as the control condition to examine the classical visual MMN effect elicited by facial stimuli (*face-only condition)*. In Condition 2 emotional voices were paired with the same facial expressions as in the face-only condition, to test the influence of concurrent vocal information on the passive processing of faces (*face-voice condition)*. In Condition 3, the *face-tone condition*, pure tone stimuli were paired with the same faces, in order to exclude the possibility that effects observed in the face-voice condition could be simply attributed to the presentation of audio-visual stimuli, regardless of their emotional meanings.

For each experimental condition, four blocks of 600 trials each were presented, including 480 standard trials (80%), 60 deviant trials (10%), and 60 target trials (10%). In Condition 1, fear faces served as standard trials and sad faces served as deviant trials in block 1 and 2, while this pattern was reversed in blocks 3 and 4 (i.e., sad faces served as standards and fear faces served as deviants). In addition to faces, 60 pictures of circles were randomly inserted in each block as *target trials* to which the participants had to press a button as response. This was to ensure that the participants were actively attending to the targets and viewing the faces passively.

In Condition 2, each face was paired with an emotional voice to construct a *bimodal face-voice condition*. Specifically, faces were paired with fearful voices in block 1 and 3, whereas faces of block 2 and 4 were paired with sad voices. This led to the four bi-modal blocks: in two blocks, incongruent pairs (*sad face-fearful voice pairs* in block 1, *fearful face-sad voice pairs* in block 4) served as standards and congruent pairs (*fearful face-voice pairs* in block 1, *sad face-voice pairs* in block 4), served as deviants; in the other two blocks, congruent pairs (*sad face-voice pairs* in block 2, *fearful face-voice pairs* in block 3) served as standards while incongruent pairs (*fearful face-sad voice pairs* in block 2, *sad face-fearful voice pairs* in block 3) served as deviants. The purpose of exchanging standard and deviant trials as congruent or incongruent pairs was to examine whether the *congruence* of face-voice pairs was relevant in evoking the vMMN. Again, 60 circles were randomly inserted as *targets* in each block.

In Condition 3, each face was paired with a pure tone of 800 ms to create a *face-tone condition*. In block 1 and 3, the faces were paired with a tone with a frequency matched with the mean f0 of the fearful voices; in block 2 and 4, the faces were paired with a tone with a frequency matched with the mean f0 of the sad voices. Sixty circles were again included as targets. Overall, for the visual stimuli, the frequency and proportion of different types of trials (standards, deviants, and targets) were identical in all three conditions; differences lied only in the accompanying auditory stimuli (see Table 1).

Note that such a visual MMN paradigm (the visual stimulus sequence consists of standards, deviants, and targets) is different from the classical aMMN paradigm in which participants would focus on the visual modality and ignore the auditory channel (Näätänen et al., 2007). This paradigm has been typically used in the visual MMN literature and it is assumed that while the classical aMMN paradigm examines the pre-attentive processing of auditory stimuli, this vMMN paradigm would tap into the *passive* perceptual processing of the deviant vs. standard visual stimuli. That is, the visual MMN component elicited in such paradigms has been considered an indication of the early passive detection of infrequent deviant information in the visual modality (Stagg et al., 2004; Susac et al., 2004; Thierry et al., 2009; Athanasopoulos et al., 2010).

### Procedure

**Figure 2:**
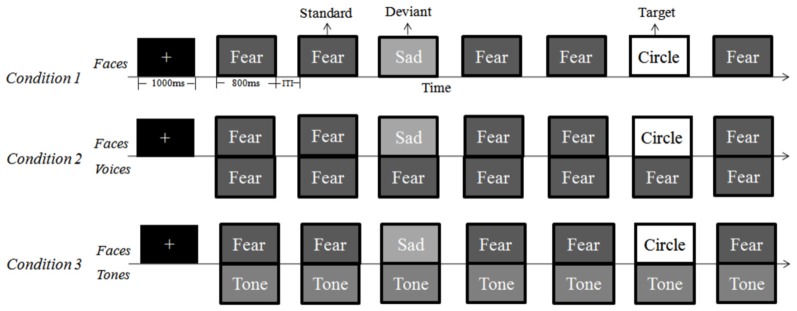
**Task procedures for each of the three conditions in the Oddball task**. In all conditions, each trial last for 800 ms; the variable inter-trial-interval varied between 500–1000 ms. In the face-voice and face-tone conditions, the visual and auditory stimuli were synchronized. From top to bottom: face-only, face-voice, face-tone.

In all three conditions, each block started with a 1000 ms fixation cross presented at the center of the monitor, followed by the sequence of trials that were presented pseudo-randomly such that two deviant trials never appeared in immediate succession, and at least three standard trials appeared in a row between two deviant ones. Each trial was presented for 800 ms, and the variable inter-trial interval was 500–1000 ms. The visual stimuli were presented at the center of the monitor and the auditory stimuli were presented binaurally via headphones at a consistent comfortable listening level. In all conditions, the participants were instructed to detect the circle targets among the faces by pressing the spacebar. For Conditions 2 and 3, in which a face was paired with a sound, it was emphasized that they should ignore the concurrent auditory stimulus (Figure 2). Each condition started with a practice block of 40 trials to familiarize participants with the procedure. The order of the four blocks within each condition and the order of the three conditions were counter-balanced among participants, and a 10-min break was inserted between blocks.

Note that the current experiment was completed by the two participant groups *before* they began the Stroop task reported by Liu et al. (2015), with an interval of at least 1 day between the two testing sessions. This decision avoided the possibility that conscious awareness of the congruence or incongruence of emotional meanings in the Stroop task (where participants had to explicitly focus on either the facial or vocal channel), would promote bias in the Oddball task, where participants were instructed to disregard the vocal/auditory channel completely and focus only on the facial stimuli.

### EEG Recording and Preprocessing

After preparation for EEG recording, participants were seated approximately 65 cm in front of a computer monitor in a dimly lit, sound-attenuated, electrically-shielded testing booth. While performing the experiment, EEG signals were recorded from 64 cap-mounted active electrodes (10/10 System) with AFz electrode as the ground, FCz electrode as on-line reference (Brain products, ActiCap, Munich), and a sampling rate of 1000 Hz. Four additional electrodes were placed for vertical and horizontal electro-oculogram recording: two at the outer canthi of eyes and two above and below the left eye. The impedance for all electrodes was kept below 5 kΩ. The EEG data were resampled off-line to 250 Hz, re-referenced to the average of all 64 electrodes, and 0.1–30 Hz band-pass filtered using EEGLab (Delorme and Makeig, 2004). The continuous data were epoched from −200 to 800 ms relative to stimulus onset with a pre-stimulus baseline of 200 ms (−200 to 0 ms). The data were then inspected visually to reject unreliable channels and trials containing large artifacts and drifts, after which EOG artifacts were rejected by means of Independent Component Analysis decomposition. For further analysis, all target trials and Standard trials that immediately followed Deviants were also excluded, leaving 480 trials (420 Standards, 60 Deviants) in each block. After artifact rejection, an average of 81.4% of data were retained for subsequent statistical analyses (Chinese face-only: 79.6%; Chinese face-voice: 81.5%; Chinese face-tone: 81.8%; English face-only: 81.7%; English face-voice: 82.3%; English face-tone: 81.3%).

### Statistical Analyses

Based on our hypothesis, the vMMN component was of sole interest in the analyses. Research indicates that MMN elicited by visual/facial stimuli is typically maximal in the occipital-parietal area in a temporal window usually covering from 100 to 300 ms after the stimulus onset (Schröger, 1998; Wei et al., 2002; Zhao and Li, 2006; Thierry et al., 2009; Athanasopoulos et al., 2010); our analyses thus focused on the same temporal and spatial regions. Visual inspection of the waveforms of grand averaged ERPs revealed more negative-going deflections elicited by Deviant trials relative to Standard ones during the 100–200 ms time window in the *occipital-parietal* region in each condition of each group, confirming our expectations. Accordingly, 14 electrodes were selected from this region (POz, PO3, PO4, PO7, PO8, PO9, PO10, Oz, O1, O2, P5, P6, P7, P8) for further analyses. For these electrodes, an exploratory investigation of the peak latency of the difference wave between Deviant and Standard trials (Deviant—Standard) yielded an averaged peak latency of 151 ms (range 143–158 ms) across conditions, consistent with previous literature and our visual inspection of the data; accordingly, the 100–200 ms time window was selected for the analysis of MMN, from which the mean amplitude values were extracted for the 14 selected electrodes.

A three-step analysis was performed on the EEG data. First, to verify whether there was a deviance effect in each condition, repeated-measures ANOVAs were conducted on the mean amplitude between 100 and 200 ms after the onset of the stimulus across selected electrodes, in each of the three conditions for each group, respectively. Specifically, in the face-only condition, *deviance* (Standard and Deviant) and *facial expression of Deviants* (fear and sadness) were adopted as within-subjects factors for a two-way repeated-measures ANOVA; in the face-voice condition, *deviance* (Standard and Deviant), *facial expression of Deviants* (fear and sadness), and *congruence of Deviants* (congruent and Incongruent) served as within-subjects factors for a three-way repeated-measures ANOVA; in the face-tone condition, *deviance* (Standard and Deviant), *facial expression of Deviants* (fear and sadness), and *tone* (frequency 1 and frequency 2) were included as within-subjects factors for a three-way repeated-measures ANOVA.

Second, *difference waves* were obtained in each block of each condition by employing an approach that has been typically used in the relevant literature, i.e., subtracting the mean amplitude of ERP responses in the Standard trials from that of the responses in the Deviant trials (Deviant—Standard) in the same block of each condition (e.g., Schröger, 1998; de Gelder et al., 1999; Wei et al., 2002; Susac et al., 2004; Zhao and Li, 2006; Astikainen and Hietanen, 2009; Thierry et al., 2009; Athanasopoulos et al., 2010). The hypothesized MMN is an early neural index of the detection of *rarity* in contrast to *regularity* in an “Oddball” sequence of stimuli (Näätänen, 1992; de Gelder et al., 1999; Susac et al., 2004; Zhao and Li, 2006; Froyen et al., 2008, 2010; Astikainen and Hietanen, 2009; Thierry et al., 2009; Athanasopoulos et al., 2010). The difference wave between the standard trials (which generated the regularity) and the deviant/odd trials (which violated the regularity and generated the rarity) in the same block (which consisted of a sequence of stimuli) could reflect such a rarity detection and was therefore calculated for each block of each condition. In this study, this calculation was conducted for the 100–200 ms time window after stimulus onset in each condition. In the *face-only* condition where only visual stimuli were presented, the difference wave reflects a pure vMMN elicited by Deviant faces relative to Standard ones (Susac et al., 2004; Zhao and Li, 2006; Astikainen and Hietanen, 2009). In the *face-voice* and *face-tone* conditions, while facial stimuli were varied as Deviant and Standard trials, the auditory stimuli were identical across all trials (fearful or sad voices in the face-voice condition; pure tones in the face-tone condition). Thus, in subtracting the ERPs in the Standards from the Deviants, potentials that were purely related with auditory processing were eliminated. The obtained difference wave, on the other hand, included potentials related with both visual processing and audio-visual interactions, which was the interest of this study. Therefore, the difference wave was considered as a component reflecting the early responses to visual stimuli with (or without) the influence of simultaneous presence of auditory cues. A similar approach of calculating and defining the MMN component has been used in previous studies for both visual MMN (Froyen et al., 2010) and aMMN (Froyen et al., 2008).

Finally, to further clarify how the difference wave was modulated by culture, a two-way repeated-measures ANOVA was conducted on the amplitude of vMMN across selected electrodes, with *Condition* (face-only, face-voice, and face-tone) as the within-subjects factor and *Group* (Chinese and English) as the between-subjects factor.

## Results

**Figure 3:**
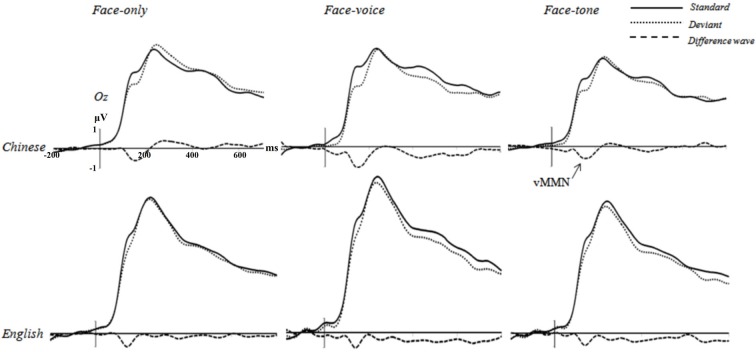
**Grand averages elicited by Standard trials (solid lines), Deviant trials (dotted lines), and the difference wave (dashed lines; Deviant—Standard) at Oz electrode for each condition of eachgroup (negative is plotted down)**.

First, the repeated-measures ANOVAs on the mean amplitude of 100–200 ms time window with *deviance, facial expression of Deviants*, *congruence of Deviants*, and* tone* as independent variables in the three conditions revealed a significant effect of *deviance* for each condition in each group (Chinese face-only condition, *F*_(1,19)_ = 7.011, *p* < 0.01, *r*^1^ = 0.519; Chinese face-voice condition, *F*_(1,19)_ = 15.256, *p* < 0.01, *r* = 0.667; Chinese face-tone condition, *F*_(1,19)_ = 6.963, *p* < 0.01, *r* = 0.518; English face-only condition, *F*_(1,18)_ = 7.709, *p* < 0.01, *r* = 0.548; English face-voice condition, *F*_(1,18)_ = 6.910, *p* < 0.01, *r* = 0.527; English face-tone condition, *F*_(1,18)_ = 6.884, *p* < 0.01, *r* = 0.526). This means that Deviant trials elicited more negative going ERP amplitude than Standard trials, implying that a visual MMN effect was evoked in each experimental condition. No significant effect involving *facial expression of Deviants*, *congruence of Deviants*, and *tone frequency* was found (*p*s > 0.37; see Figure 3).^2^

**Figure 4:**
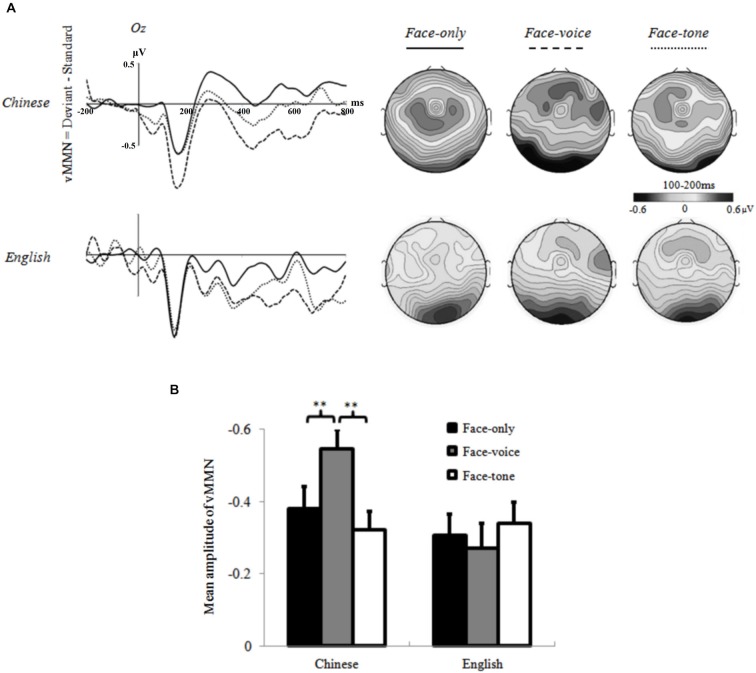
**(A)** Grand averages at Oz electrode and topographic maps of vMMN (100–200 ms) for each condition of each group; **(B)** Mean amplitude values of vMMN averaged across selected electrodes for each condition of each group. **: *p* < 0.05.

The subsequent two-way repeated measures ANOVA on vMMN with *Condition* (face-only, face-voice, and face-tone) and *Group* (Chinese and English) as two factors revealed a significant main effect of *Condition* (*F*_(2,70)_ = 34.319, *p* < 0.01, *r* = 0.573). Overall, a larger vMMN was observed in the face-voice condition than the other two conditions. Of even greater interest to our hypotheses, the interaction of *Condition* and *Group* was significant (*F*_(2,70)_ = 5.829, *p* < 0.05, *r* = 0.277). Simple effect analysis specified that the effect of *Condition* was significant in the Chinese group (*F*_(2,34)_ = 6.493, *p* < 0.01, *r* = 0.399), who showed a larger vMMN in the face-voice condition than the face-only and face-tone conditions. No such effect was observed in the English group (*p* = 0.32). The effect of *Group* was significant in the face-voice condition, where the Chinese showed a larger vMMN than the English group (*F*_(2,34)_ = 6.302, *p* < 0.01, *r* = 0.395; see Figure 4).

We also analyzed the MMN data with equal number of Standards (those preceding the Deviants) and Deviants and consistent results were found. In the first ANOVA, the main effect of *deviance* was significant on the mean amplitude of 100–200 ms time window across the selected electrodes for each condition of each group (Chinese face-only condition, *F*_(1,19)_ = 6.717, *p* < 0.01, *r* = 0.511; Chinese face-voice condition, *F*_(1,19)_ = 13.046, *p* < 0.01, *r* = 0.638; Chinese face-tone condition, *F*_(1,19)_ = 6.352, *p* < 0.01, *r* = 0.501; English face-only condition, *F*_(1,18)_ = 5.513, *p* < 0.05, *r* = 0.484; English face-voice condition, *F*_(1,18)_ = 7.106, *p* < 0.01, *r* = 0.532; English face-tone condition, *F*_(1,18)_ = 6.141, *p* < 0.01, *r* = 0.504). No significant effect involving *facial expression of Deviants*, *congruence of Deviants*, or *tone frequency* was found (*p*s > 0.20). The second ANOVA on vMMN revealed a significant main effect of *Condition* (*F*_(2,70)_ = 23.774, *p* < 0.01, *r* = 0.504) and a significant interaction of *Condition* and *Group* (*F*_(2,70)_ = 5.701, *p* < 0.05, *r* = 0.274). Simple effect analysis specified that the effect of condition was significant in the Chinese group (*F*_(2,34)_ = 5.079, *p* < 0.05, *r* = 0.361), who showed a larger vMMN in the face-voice condition than the face-only and face-tone conditions. No such effect was observed in the English group (*p* = 0.21).

## Discussion

To our knowledge, this is one of the first studies to explore cultural differences in the passive on-line processing of multi-sensory emotional cues from faces and voices, by comparing “East vs. West” (Chinese vs. English North Americans) in an Oddball-like task. Our research provides solid evidence in support of our hypotheses as cultural background robustly modulated the vMMN component in distinct ways. In particular, the Chinese group exhibited a larger vMMN component in the face-voice condition relative to the other two conditions, whereas no such a pattern was witnessed in the English group. This suggests that Chinese participants were more influenced by concurrent vocal cues than English participants, an effect observed as early as 100 ms after stimulus onset as participants passively decoded emotion from conjoined facial and vocal expressions. More broadly, these patterns fit with the idea that individuals from East Asian cultures are more sensitive to vocal cues in communication (Kitayama and Ishii, 2002; Ishii et al., 2003; Tanaka et al., 2010; Liu et al., 2015).

As expected, a visual MMN component was observed in each experimental condition for each group, indicated by a significant effect of *deviance* (i.e., difference wave between the more negative going potentials in Deviant vs. Standard trials). This difference wave is considered a visually-related component reflecting responses to visual stimuli in the presence of ignored auditory cues. The observation of vMMN in all three conditions suggests that both groups detected the infrequent changes in facial expressions at an early temporal stage, even though they were only passively viewing faces as they watched for visual targets (a circle). More interestingly, while in the literature vMMN was mostly reported in the processing of physical properties or simple semantic meanings (e.g., facial expressions; Susac et al., 2004; Zhao and Li, 2006; Astikainen and Hietanen, 2009), here, the MMN observed in the face-voice condition suggests that the regularity/rarity contrast defined by more complex meaning conjunctions (congruent vs. Incongruent face-voice pairs) could also effectively evoke such a component (Althen et al., 2011). In addition, no significant effects involving facial expression, face-voice congruence, or tone frequency were found on the 100–200 ms time window, indicating that exchanging the standard and deviant trials did not influence the MMN component (e.g., comparable MMN was observed in Block 1 and Block 3 of Condition 2; see Table 2). This suggests that the difference wave observed between Standards and Deviants was not determined by the difference in physical properties of the two types of trials, but rather due to the abstraction of the regularity/rarity contrast generated by the frequent/infrequent presentation of standard/deviant trials in the same stimuli sequence in each block. This also implies that a significant MMN effect was constantly observed no matter what specific emotional properties or property combinations defined the regularity/rarity of the stimulus series, conforming to the established role of MMN in the literature as an index of early, pre-semantic rarity detection independent of what is being processed (Stagg et al., 2004; Maekawa et al., 2005; Schirmer et al., 2005; Pöppel, 2009).

**Table 2:** **Types of standard and deviant trials of each block in each condition**.

	Condition 1		Condition 2			Condition 3	
	Face-only		Face-*Voice*			Face-*Tone*	
	Standard	Deviant	Standard	Deviant	Congruence of deviant	Standard	Deviant
Block 1	**Sad**	**Fear**	**Sad**-*Fear*	**Fear**-*Fear*	Congruent	**Sad**-*Tone 1*	**Fear**-*Tone 1*
Block 2	**Sad**	**Fear**	**Sad**-*Sad*	**Fear**-*Sad*	Incongruent	**Sad**-*Tone 2*	**Fear**-*Tone 2*
Block 3	**Fear**	**Sad**	**Fear**-*Fear*	**Sad**-*Fear*	Incongruent	**Fear**-*Tone 1*	**Sad**-*Tone 1*
Block 4	**Fear**	**Sad**	**Fear**-*Sad*	**Sad**-*Sad*	Congruent	**Fear**-*Tone 2*	**Sad**-*Tone 2*

*Facial stimuli (in bold) of each block are identical across three conditions; auditory stimuli (in italic) are identical across standard and deviant trials within each block. In each block of each condition, MMN was calculated by subtracting the ERP responses to Standards from that to the Deviants in the same block. i.e., in each block, MMN = Deviant (10%)—Standard (80%)*.

Of greater theoretical importance, our experiment supplies initial evidence that the cultural background of participants modulates emotion perception even during the early, pre-semantic temporal stage of stimulus processing (Pöppel, 2009). Specifically, individuals in the Chinese group showed larger vMMN amplitudes in the face-voice condition than the face-only and face-tone conditions, whereas no difference was observed across conditions for English participants. Larger amplitude of the MMN component is thought to reflect the greater magnitude of the detected discrepancy of the Deviant stimulus (Campbell et al., 2007); therefore, the larger vMMN of the Chinese group in the face-voice condition suggests that Chinese participants detected larger deviancy in this condition than in the other two conditions. Given that facial expressions in all conditions were identical and evoked comparable visual potentials, it can be inferred that the larger MMN effect in the face-voice condition is due to the presence of concurrent vocal information as well as the interaction that occurred between facial and vocal stimuli. In other words, Chinese participants may have involuntarily integrated the accompanying (to-be-ignored) vocal information while passively processing the facial expressions, which enhanced their MMN effect, whereas this did not occur for the English group. These findings establish that Chinese participants are more sensitive to vocal cues compared to English participants even at early temporal stages of emotion processing that are presumably mostly outside of attentional focus and control, indexed by the vMMN component.

Interestingly, no difference was found in the face-tone condition compared with the face-only condition. This suggests that the effects we observed are unique to *human* vocal expressions that bear special significance to communication and person perception (Belin et al., 2000). This is compatible with species-specific effects on the integrative perception of cross-channel cues observed in previous literature, which, for example, reported that the recognition of emotional body posture was influenced by human vocalizations to a larger extent than by animal sounds (Van den Stock et al., 2008). Another possible reason that the face-only and face-tone conditions yielded similar results is that, compared to the face-voice condition, the other two conditions are more similar to each other. While the vocal stimuli in the face-voice condition consisted of a variety of different utterances, auditory stimuli in the face-tone condition (i.e., a single pure tone) were identical across trials, which may have allowed participants to rapidly habituate to these unchanging stimuli in the face-tone condition similar to the face-only condition. Future studies using non-vocal auditory stimuli with similar degree of variety and complexity to the vocal stimuli (e.g., environmental sounds) might help to clarify this question.

In the English group, no difference in vMMN was found between the face-voice condition and the other two conditions; i.e., while the MMN effect was enhanced by simultaneous vocal cues in the face-voice condition for the Chinese participants, similar evidence was not observed in the English group. Interestingly, a previous study reported that an aMMN component was induced by infrequent discrepant information in concurrent facial cues for Dutch participants (de Gelder et al., 1999). This finding, coupled with our results, implies an asymmetric pattern in Western participants, whereby facial displays automatically modulate the aMMN (i.e., early unattended processing of vocal cues) but the evidence that vocal cues automatically influence the vMMN (passive facial expression processing) was absent. A similar asymmetry was documented in letter-speech sound processing in Dutch participants, where the aMMN in response to speech-sounds was modulated by concurrent visual letters, whereas the evidence of the vMMN in response to letters influenced by concurrent speech-sounds was not found (Froyen et al., 2008, 2010). Given the fact that these findings were all observed in participants from the Western culture (English North Americans and Dutch), this asymmetric pattern in the MMN effect, showing that Western participants were influenced by faces but lacking evidence that they were affected by voices, is in keeping with our previous findings based on analyses of N400 and behavioral accuracy data (Liu et al., 2015). More generally, they also fit with the culture-specific hypothesis that Westerners possess a higher sensitivity to facial cues than vocal information when compared to East Asians (e.g., Tanaka et al., 2010).

It is worth underscoring that our findings demonstrate that the effect of cultural origin on multi-sensory emotion perception occurs particularly early after stimulus onset. Indeed, other socio-cultural factors are known to impact emotion processing at a very early stage. For instance, effects of race on facial expression processing have been observed as early as the N170 component; compared to inverted other-race faces, inverted same-race faces lead to greater recognition impairment and elicit larger and later N170 amplitudes (Gajewski et al., 2008; Vizioli et al., 2010). Similarly, facial expressions embedded in backgrounds of fearful social scenes (e.g., a car accident) elicited larger N170 than faces in happy and neutral scenes, suggesting that the early structural processing of emotional faces is influenced by concurrent contextual information in the visual modality (Righart and de Gelder, 2008). Coupled with our results, these findings imply that various cultural and social factors related to our experiences during development and through socialization are likely to play an important role, with seemingly rapid effects, on the processing of emotional stimuli.

As mentioned, it has been shown that the vMMN component is modulated by linguistic background of the participants during color perception (Thierry et al., 2009); the present study provides the first evidence that this component is also sensitive to the participants’ *cultural* background in the domain of audio-visual emotion perception, and broadens the knowledge of the role of culture in perception and cognition in general. Together with our previous evidence that cultural origin affects N400 responses and behavioral accuracy for the same participants when consciously attending to facial-vocal emotions (Liu et al., 2015), the current data paint a bigger picture of the role of culture in different aspects and temporal stages of multisensory emotion processing. In our previous study using a Stroop-like paradigm (Liu et al., 2015), while important cultural differences were noted and there was clear evidence that English participants are more attuned to facial expressions than Chinese, we did not uncover direct behavioral or N400 evidence showing that the Chinese participants were more sensitive to vocal cues than English participants as predicted (i.e., the Chinese did not show a significant differential bias between face and voice attention conditions, perhaps due to methodological factors, see Liu et al., 2015, for details). However, current examination of the vMMN clearly demonstrates the predicted higher sensitivity to vocal cues of the Chinese during an earlier temporal window of passive emotion processing, elaborating upon our observation that English participants are more attuned to faces under different task conditions (Liu et al., 2015). When put together, these two studies argue that cultural origin plays a significant role at both earlier and later stages of multi-sensory emotion processing, which promoted the higher sensitivity to vocal cues of the Chinese group during the earlier MMN processing stage, and the higher susceptibility to facial cues of the English group during the later N400 and behavioral processing stage. This demonstrates the robust influence of cultural origin on the processes for appraising and interpreting emotional expressions during communication; in particular, this cultural effect appears at a very early stage shortly after the onset of the emotional stimuli (100–200 ms), continues to the semantic processing stage (around 400 ms), and finally affects the explicit behavioral performance in perceiving emotions, compatible with the processing patterns proposed by existing models (Schirmer and Kotz, 2006).

Our claim that Chinese participants are more attuned to information in the vocal communication channel based on on-line neurophysiological measures is consistent with previous arguments of a similar behavioral bias for vocal emotions over faces for Japanese participants (Tanaka et al., 2010). Our results are also in line with observations in non-emotional communication that Japanese speakers use visual cues less than English speakers when interpreting audiovisual speech (Sekiyama and Tohkura, 1991). As discussed in the Introduction Section, these culture-specific biases in communication are arguably the product of acquired display rules that regulate how people should communicate their feelings in a socially-appropriate manner in a specific culture (Park and Huang, 2010; Engelmann and Pogosyan, 2013). Hypothetically, culturally-reinforced practices that promote restrained facial expressions and reduced eye contact in East Asian collectivist cultures, meant to avoid conflict and to maintain social harmony, limit the availability of visual facial cues for these cultures, meaning that greater perceptual weight would be accorded to vocal information during communication (Tanaka et al., 2010; Liu et al., 2015). These ideas are ripe for further testing. In addition, while this study focused on in-group emotion perception, using out-group stimuli in future studies would help to determine whether the observed cultural differences in the current study, which were arguably motivated by display rules, would transfer to another language and culture (see Elfenbein, 2013, for a related view). That is, would culture-specific neural responses observed here persist when participants are presented *out-group* stimuli that reflect the cultural norms of a foreign culture? This would be an interesting question for future work. Another possible future direction is to pinpoint the brain generators of the observed cultural effects by using localization approaches (e.g., fMRI) would help. For instance, cultural differences during facial expression processing appear to modulate activation of the amygdala (Moriguchi et al., 2005; Chiao et al., 2008; Derntl et al., 2009, 2012; Adams et al., 2010), a structure associated with rapid selective processing of emotion-related information independent of attention and consciousness (Morris et al., 1996; Pessoa, 2010). It will be useful to test whether culture-specific patterns affecting the early pre-semantic stage of emotional processing, such as the vMMN observed here, can also be elucidated in the spatial dimension by future work that focuses on how functional brain networks are modulated by cultural experiences.

In addition to effects of cultural *origin*, cultural *immersion* represents another case where cross-cultural communication can be hampered by display rules or other forms of acquired knowledge governing inter-personal communication. Individuals who live for extended periods in a foreign culture show more similar (neuro)cognitive patterns to their host culture in various cognitive domains, including facial expression perception (Derntl et al., 2009, 2012; Damjanovic et al., 2013) among others (Athanasopoulos, 2009; Athanasopoulos et al., 2010). In light of differences in how Chinese and English process multisensory emotional stimuli, how would cultural immersion and exposure to a new set of social conventions impact on these patterns, for example, in the case of Chinese immigrants living in North America? We are now exploring this question in a follow-up study (Liu et al., in review) as a new step to advance knowledge of the role of culture in emotional communication.

## Conflict of Interest Statement

The authors declare that the research was conducted in the absence of any commercial or financial relationships that could be construed as a potential conflict of interest.

## Footnotes

^1^Effect size, calculated as $r=\surd F/(F+df_{\text{error}})$ (Rosnow and Rosenthal, 2003).

^2^We also ran the ANOVAs including *laterality* (*left: PO3, PO7, PO9, O1, P5, P7; middle: POz, Oz; right: PO4, PO8, PO10, O2, P6, P8*) as another independent variable in each condition of each group. While the main effect of *deviance* was again found to be significant in each condition (*p*s < 0.05), no effect involving *laterality* was found (*p*s > 0.13). This factor was therefore not included for the subsequent analysis.
